# A new *Haniffia* species (Zingiberaceae) and a new generic record from Sarawak, Malaysian Borneo

**DOI:** 10.1186/s40529-014-0051-9

**Published:** 2014-06-05

**Authors:** Sin Yeng Wong, Im Hin Ooi, Peter C Boyce

**Affiliations:** 1 Department of Plant Science and Environmental Ecology, Faculty of Resource Science and Technology, Universiti Malaysia Sarawak, Kota Samarahan, 94300 Sarawak Malaysia; 2 Institute of Biodiversity and Environmental Conservation, Universiti Malaysia Sarawak, Kota Samarahan, 94300 Sarawak Malaysia

**Keywords:** Haniffia santubongensis, Mount Santubong, Phylogeny, Taxonomy

## Abstract

**Background:**

*Haniffia* Holttum is a genus of three described species of terrestrial gingers hitherto restricted to Peninsular Thailand and various localities in Peninsular Malaysia.

**Results:**

With generic placement confirmed using nrITS, *trn* K and *mat* K plastid sequence data, *Haniffia santubongensis* S.Y. Wong & P.C. Boyce is described as a taxonomically novel species representing a new generic record for Borneo, to where it is endemic to Mount Santubong, Kuching Division, NW Sarawak, Malaysian Borneo. An identification key to all species is given and *H. santubongensis* is illustrated from living plants.

**Conclusion:**

*Haniffia santubongensis* is the fourth species of *Haniffia* so far described, and the first occurring on sandstone.

**Electronic supplementary material:**

The online version of this article (doi:10.1186/s40529-014-0051-9) contains supplementary material, which is available to authorized users.

## Background

*Haniffia* Holttum is a genus of three described species of terrestrial gingers hitherto restricted to Peninsular Thailand and various localities in Peninsular Malaysia. The three described species are all seemingly locally endemic. The type species, *H. cyanescens* (Ridl.) Holttum, is restricted to Bukit Tanga (Negeri Sembilan, Peninsular Malaysia), with a variety, *H. cyanescens* var. *penangiana* C.K. Lim, occurring on Pulau Pinang and Kedah. The most recently recognized species, *H. flavescens* Y.Y. Sam & Julius (Sam et al. [2009]) is known only from Endau Rompin National Park (Johor, Peninsular Malaysia). The sole extra-Malaysian species, *H. albiflora* K. Larsen & Mood, is confirmed only from Nam Tok Chatwarin, Naratiwat, Thailand. A summary of the taxonomic history of *Haniffia* Holttum is presented by Larsen and Mood ([2000]).

## Methods

### Plant material

Fresh leaf material of *Haniffia santubongensis* was collected from the type locality, Mount Santubong. The type specimen with the spirit material was deposited to SAR.

#### DNA extraction, amplification and sequencing

Genomic DNA was extracted using a modified CTAB protocol. ITS, *trn* K intron and *mat* K gene were amplified using the same set of primers as in Leong-Škorničková et al. ([2011]). PCR products were purified using GenJet PCR purification kit (Thermo Scientific, Vilnius, Lithuania) and sent for sequencing in forward and reverse directions at First BASE Laboratories Sdn. Bhd., Selangor, Malaysia. Sequences were edited, assembled and aligned using MUSCLE (Edgar [2004]) as implemented in Geneious Pro v5.6.4 (Biomatters Ltd., Auckland, New Zealand; http://www.geneious.com; Drummond et al. [2012]). Two newly generated sequences were deposited into GenBank under accession numbers KJ452785 (*trn* K/*mat* K) and KJ452784 (ITS), and combined with sequences included in Leong-Škorničková et al. ([2011]). When the placement of the new sequences was confirmed to fall within the *Kaempferia* Clade, then the final data matrix was reduced to include all the species in the *Kaempferia* Clade with *Cautleya gracilis* (Sm.) Dandy and *Roscoea cautleoides* Gagnep. selected as outgroups. Table 1 shows the list of species included for the final data matrix. The data matrix was deposited into TreeBASE (reviewer access URL: http://purl.org/phylo/treebase/phylows/study/TB2:S15361?x-access-code=f78126f9da891d3c6999dd52dfafdf77&format=html).

**Table 1:** **List of species included in this study with vouchers (herbarium location) and GenBank accession numbers for DNA sequences used in the phylogenetic analyses**

Taxon	Voucher	***trn***K (including***mat***K)	ITS
*Haniffia albiflora* K. Larsen & Mood	Kress #99-6370 (US)	AF478855	AF478756
*Haniffia cyanescens (* Ridl.) Holttum	Julius et al. FRI56069	JF825538	JF825533
*Haniffia flavescens* Y.Y. Sam & Julius	Julius et al. FRI57598	JF825539	JF825534
*Haniffia santubongensis* S.Y. Wong & P.C. Boyce	P.C. Boyce & S.Y. Wong ZI22	KJ452785	KJ452784
*Newmania orthostachys* N.S. Lý & Škorničk.	Lý 470 (VNM, E, P, SING)	JF825540	JF825537
*Newmania serpens* N.S. Lý & Škorničk.	Lý 332 (VNM, E, P, SING)	JF825541	JF825536

### Phylogenetic analyses

Phylogenetic analyses were performed with PAUP*4.0b10 (Swofford [2002]) for maximum parsimony (MP) reconstruction with all characters equally weighted. The most parsimonious trees were obtained with heuristic searches of 1,000 replicates with random stepwise sequences addition, tree bisection-reconnection (TBR) branch swapping, collapse of zero-length branches, with the multiple-tree option in effect, and saving up to 10,000 trees from each random sequence addition.

The most suitable nucleotide substitution model for each of the gene regions was selected in jModeltest ver. 0.1.1 (Posada [2008]) using Akaike information criterion (AIC). General time reversible (GTR + I + G) was the nucleotide substitution model selected. Maximum likelihood (ML) analyses were carried out using RAxML 7.2.6 (Stamatakis et al. [2008]). Maximum likelihood bootstrap values were obtained by running 10,000 replicates. Bayesian phylogenetic analyses were performed with MrBayes ver. 3.1.2 (Huelsenbeck and Ronquist [2001]). Markov chain Monte Carlo (MCMC) was repeated twice to assure parameter convergence. The MCMC algorithm was run for 2,000,000 generations with one cold and three heated chains, starting from random trees and sampling one out of every 100 generations. Convergence was assessed by using the standard deviation of split frequencies as convergence index with values < 0.005 interpreted as indicating good convergence. The first 10% of trees were discarded as burn-in. Remaining trees were used to construct 50% majority-rule consensus trees.

## Results and discussion

### Morphology and biogeography

In overall appearance *H. santubongensis* is most similar to *H. cyanescens*, sharing with that species a wide bluish labellum and semi-glossy fruits. However, *H. santubongensis* is clearly distinct from *H. cyanescens* by the lateral staminodes with an oblique bifid tip, the labellum distally notched (not deeply divided), and differences in labellum colouration and patterning (most notably the presence of a median yellow callus in *H. santubongensis*).

*Haniffia santubongensis* represents a new generic record for Borneo where it is locally endemic to Gunung Santubong. Combined with the three locally endemic species in Peninsular Malaysia and P. Thailand, it provides further compelling evidence that these *Haniffia* species, along with numerous other examples in families as diverse as the aroids, the palms, Rubiaceae Juss., and the genus *Hanguana* Blume, represent relictual fragments of the Riau Pocket phytochore (Ashton [2005]; Corner [1960]).

### Molecular analyses

**Figure 1:**
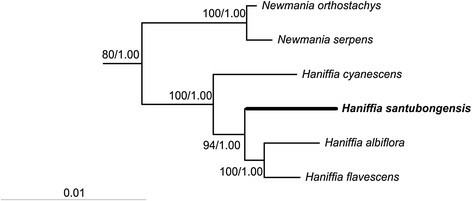
**Molecular phylogeny of the** ***Kaempferia*** **Clade (Zingiberaceae) indicating the placement of** ***Haniffia santubongensis*** **in the combined** ***trn*** **K/ITS analysis.** Tree length = 633 steps, Consistency Index = 0.7615, Retention Index = 0.7729, Rescaled Consistency Index = 0.5886. Bootstrap numbers from Maximum Likelihood and Posterior Probabilities are provided on the branch.

The combined ITS-plastid dataset contained the new species and 19 species in the *Kaempferia* Clade recognized in Leong-Škorničková et al. ([2011]) together with two outgroup species. The dataset comprises 2,115 characters: 1,682 characters were constant, 251 variable, but parsimony uninformative, and 182 (8.6%) were parsimony informative. The data analyses produced 32 shortest trees of length 633 steps with a Consistency Index = 0.7615 and Retention Index = 0.7729. The tree topologies obtained from three analyses are similar and consistent with the results from Leong-Škorničková et al. ([2011]). Figure 1 shows the partial tree obtained from maximum likelihood analysis. The new species is shown to be a sister taxon to *H. albiflora* and *H. flavescens. Haniffia cyanescens* is supported basally to the three *Haniffia* species. The new species of *Haniffia* differs by nine nucleotide substitutions (two in *trn* K intron and seven in ITS), an addition of 17 bps (*trn* K intron), an eight bp substitution (ITS), and seven bp deletion (ITS).

### Taxonomic treatment

#### Key to Haniffia species

1a. Corolla lobes and staminodes pale yellow; labellum pale yellow with golden yellow median band, apex emarginate. . . . . . . . ***H. flavescens***

1b. Corolla lobes and staminodes white; labellum white, white with purple veins or dark blue-violet; apex bilobed. . . . . . . . . 2

2a. Leaf blade 10–14?×?2–3.5 cm; ligule with long white hairs; flowers 2–5 in each inflorescence. S Thailand. . . . . . . . . ***H. albiflora***

2b. Leaf blade 17–21 × 3–4.9 cm; ligule glabrous or sparsely pubescent; flowers 5–7 in each inflorescence. Malaysia. . . . . . . . . 3

3a. Lateral staminodes bifid at apex; labellum distally notched, with a central yellow median band. NW Borneo. . . . . . . . ***H. santubongensis***

3b. Lateral staminodes not bifid; labellum deeply spit, without a central yellow median band. Peninsular Malaysia. . . . . . . . 4

4a. Labellum dark blue-violet with white veins; lateral staminodes obovate to oblanceolate. . . . . . . . ***H. cyanescens*** **var.** ***cyanescens***

4b. Labellum white with violet veins; lateral staminodes lanceolate. . . . . . . . . . ***H. cyanescens*** **var.** ***penangiana***

**Figure 2:**
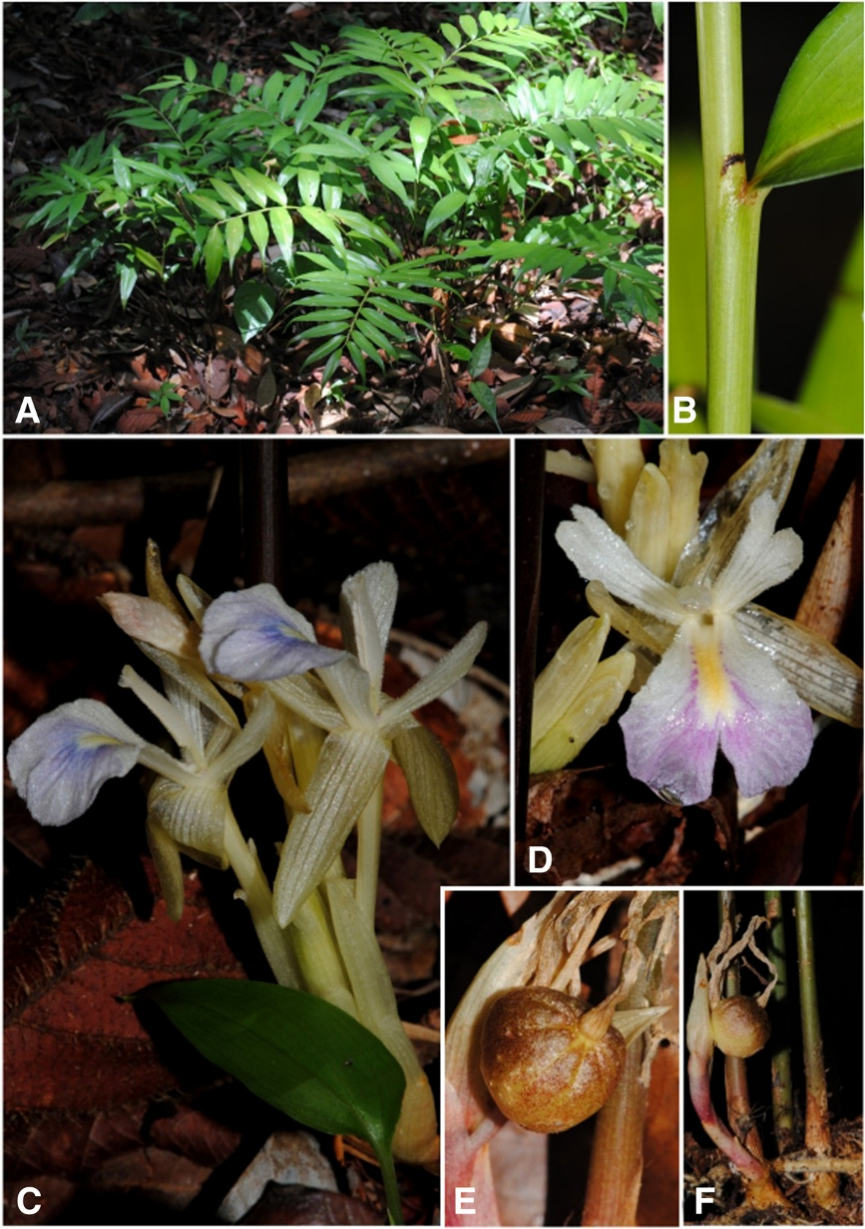
***Haniffia santubongensis*** **S.Y. Wong & P. C. Boyce. A**. Plant in habitat. **B**. Detail of ligule; note the blackish colour of the free portion. **C**. Inflorescence. **D**. Single flower; note the bifid tips to the lateral staminodes and the yellow callus in the middle of the labellum. **E**. Infructescence. **F**. Detail of single fruit; note the semi-glossy slightly warty surface, and the prominent floral remains.

***Haniffia santubongensis*** S.Y. Wong & P.C. Boyce, sp. nov. — Type: Malaysia, Sarawak, Kuching, Gunung Santubong, Summit Trail, just after F4, 04 44 12.1 N 110 19 30.6 E, 2 Sept. 2005, *P.C. Boyce & S.Y. Wong ZI-22* (holotype: SAR; isotype: SAR (spirit), FRIM (spirit)) Figure 2.

### Diagnosis

*Haniffia santubongensis* most closely resembles *H. cyanescens* var. *cyanescens* (Negeri Sembilan) but is readily distinguished by the oblique-tipped, bifid lateral staminodes, and labellum distally notched (not deeply split) with a yellow median band extending from the entrance to the basal spur to the innermost extent of the lip distal division.

### Description

Terrestrial, clumping herb ca 50 cm tall; *rhizomes* ca 10 mm diam., shallowly buried in soil; roots fibrous. *Leafy pseudostem* 5–25 per clump, closely spaced, composed of leafless sheaths and leaf sheaths, base ca 1 cm diam.; *leafless sheaths* ca 6 per shoot, occupying the lower 1/3-1/2, reddish, glabrous, membranous on apex and margins. *Leaves* 10*–* 20 per shoot, sub-sessile, sheaths very short, to ca 1 cm long; *ligule* obtuse to almost truncate, slightly emarginate, ca 3 mm long, soon turning black, glabrous; *petiole* reddish; *lamina* narrowly elliptic to lanceolate, 8.5-15 × 2.5-3 cm, base cuneate, apex acuminate, acumen 6–20 mm long, glabrous, bright green, abaxially very slightly paler with deep green slender primary lateral veins. *Inflorescences* borne at base of leafy shoot, usually solitary, rarely two together, 5–7 cm long, with ca 5 flowers per inflorescence, peduncle to 3.5 cm long, reddish where exposed, otherwise pale green, basal sheaths 1–2, 1–2 cm, pink. *Bracts* boat-shaped, 15–25 × 6–10 mm, spirally arranged, membranous, reddish green, glabrous, each subtending one flower. *Calyx* tubular, 20–30 mm long, apex unequally tridentate, greenish, translucent, hairy at tip. *Floral tube* 36–70 mm long, greenish white, slightly glandular-hairy inside and out; *dorsal corolla lobe* elliptic, 20–26 × ca. 6 mm, membranous, greenish white with darker veins, *lateral corolla lobes* narrowly elliptic, 20–25 × ca 4 mm, membranous, greenish white with darker veins, glabrous. *Lateral staminodes* spatulate, apex oblique bifid, 15–20 × 4–6 mm, greenish white with darker veins, both surfaces covered with dense, greenish white glandular hairs. *Labellum* obovate, 21–25 × 10–14 mm, swollen at base where adnate to staminodes, apex distally notched, distal lobes curved downwards, white with blue staining, lobes suffused purple or dark blue-violet, centrally with a yellow median callus, upper surface covered with dense, white glandular hairs, lower surface sub-glabrous. *Stamen* white, covered with dense, glandular hairs; filament 5–6 mm long; anther 6–10 mm long; anther crest 2–2.5 mm long, apex bidentate, white. *Style* 46–54 mm long, glabrous; stigma cup-shaped, ca1.8 mm long, ciliate. *Ovary* 3-locular, ovules many. *Epigynous glands* two, free, linear, 2–2.4 mm long, yellow. *Fruit* a capsule, dehiscent, somewhat 3-sided, globose with prominent terminal floral remains, ca. 2 × 2 cm, semi-glossy brownish, heavily stained with reddish brown speckles on a cream base, exterior very sparsely warty, splitting longitudinally into 3 valves, valves fleshy, usually with 3-locules well developed. *Seeds* ellipsoid-obovoid, 6–7 × 3–4 mm, glossy brown, turning greyish-black, aril thick, white when fresh.

### Ecology

Partially shaded, deep sandy peat podzols of ridge kerangas in *Dryobalanops*-dominated hill forest; ca 200–250 m asl.

### Distribution

*Haniffia santubongensis* is known only from the type locality where it occurs as two separate, dense, populations.

### Etymology

The species epithet is derived from the name of the type locality.

## Conclusion

*Haniffia santubongensis* is the fourth species of *Haniffia* and represents a new generic record for Borneo.

## Authors’ original submitted files for images

Below are the links to the authors’ original submitted files for images. Authors’ original file for figure 1 Authors’ original file for figure 2
