# Supplementary figures and images for: A new Haniffia species (Zingiberaceae) and a new generic record from Sarawak, Malaysian Borneo
Source: Bot Stud. 2014 Jun 5;55:51. doi: 10.1186/s40529-014-0051-9 (PMC5432771; doi:10.1186/s40529-014-0051-9)

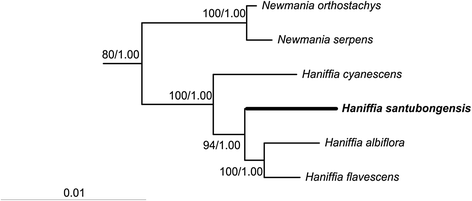

Supplement: Supplementary file 1 — Authors’ original file for figure 1 [file 40529_2014_51_MOESM1_ESM.gif]

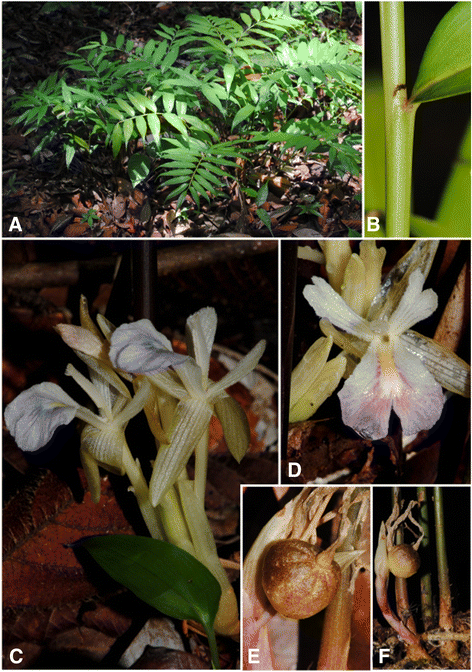

Supplement: Supplementary file 2 — Authors’ original file for figure 2 [file 40529_2014_51_MOESM2_ESM.gif]
